# Implementation of a Universal School-Based Child Sexual Abuse Prevention Program: A Longitudinal Cohort Study

**DOI:** 10.1177/08862605231158765

**Published:** 2023-03-03

**Authors:** Kate Guastaferro, Stacey L. Shipe, Christian M. Connell, Elizabeth J. Letourneau, Jennie G. Noll

**Affiliations:** 1 The Pennsylvania State University, University Park, USA; 2 New York University, USA; 3 State University of New York – Binghamton University, USA; 4 Johns Hopkins University, Baltimore, MD, USA

**Keywords:** child sexual abuse, prevention, implementation, reach, effectiveness

## Abstract

Child sexual abuse (CSA) is a public health problem of considerable magnitude. The prevailing primary prevention strategies are universal, school-based CSA prevention programs, some of which have been designated as evidence-based, such as *Safe Touches*. However, to reach their public health impact potential, effective universal school-based CSA prevention programs require effective and efficient dissemination and implementation strategies. The purpose of this study was to demonstrate the reach and effectiveness of a school-based CSA prevention curriculum, *Safe Touches*, when implemented on a wide scale. Using a longitudinal cohort design, children in second grade classrooms in public elementary schools in five counties received the *Safe Touches* workshop and completed surveys designed to assess gains in knowledge at four timepoints (one week prior, immediately post-workshop, 6- and 12-months post-workshop). In total, the *Safe Touches* workshop was delivered in 718 classrooms in 92% of school districts, reaching ~14,235 second graders. Multilevel models (*n* = 3,673) revealed that *Safe Touches* significantly increased CSA-related knowledge, and that these gains were maintained 12-months post-workshop (*p*s < .001). There were some small but significant time-varying effects among participants in schools with a greater percentage of low income and minority students, but these effects largely disappeared 12-months post workshop. This study demonstrates that a single-session, universal school-based CSA prevention program can effectively increase children’s knowledge when implemented and disseminated on a wide scale and knowledge gains can be retained 12-months post intervention.

Child sexual abuse (CSA) is a global public health problem of considerable magnitude impacting 12% of children under 18 (Barth et al., 2013). Official reports made to the child protective service (CPS) system conservatively suggest rates of CSA in the United States equal roughly 61,000 children annually (U.S. Department of Health & Human Services Administration for Children and Families Administration on Children Youth and Families Children’s Bureau, 2021). Meta-analyses and systematic reviews showcase the relationship between CSA and lifelong sequelae (Maniglio, 2009) including physical (Irish et al., 2010), psychological (Amado et al., 2015; Maniglio, 2010, 2013; Noll, 2021), and behavioral health outcomes such as adolescent pregnancy (Noll et al., 2009) and revictimization (Walker et al., 2019). Accounting for corresponding healthcare costs and loss of productivity, the estimated lifetime economic burden of CSA is estimated to exceed $9.3 billion (Letourneau et al., 2018). Given the scope, gravity, and consequences associated with CSA, the primary prevention of CSA is of critical public health importance.

The prevailing primary prevention strategy in the United States is child-focused programs delivered in schools targeting elementary school-age children (Walsh et al., 2018). Broadly, school-based programs focus on strengthening children’s knowledge of protective skills to prevent victimization and improving children’s awareness and use of preventive strategies (Finkelhor, 2009). Meta-analyses and systematic reviews suggest that universal school-based strategies have demonstrated effectiveness in improving children’s CSA-related knowledge (Davis & Gidycz, 2000; Walsh et al., 2018; Zwi et al., 2007). For example, the “*Who Do You Tell*?” program significantly increases children’s knowledge related to inappropriate and appropriate touch (Tutty, 1997). Tutty et al. (2020) compiled outcomes for 6,198 students across 50 schools in Calgary, Canada that had received the program 1 to 3 times over 8 years. Findings demonstrated that CSA knowledge and attitudes significantly improved and were maintained, with the largest effect shown in second graders (*effect size* = 0.76). Another program, *Safe Touches*, developed by the New York Society for the Prevention of Cruelty to Children, demonstrated a significant increase in knowledge of inappropriate touches among students in a cluster randomized trial that received the workshop compared to those who did not (Holloway & Pulido, 2018; Pulido et al., 2015). A decline in rates of CSA has not been directly associated with participation in school-based prevention programs; however, it is likely that CSA prevention facilitates disclosures (Finkelhor, 2007; Gibson & Leitenberg, 2000). For example, a study of children undergoing forensic interviewing who had participated in a school-based prevention program were 82% more likely to disclose than their peers who did not participate in the program (Elfreich et al., 2020). This should be interpreted with caution as the children were undergoing a forensic interview which only occurs if abuse is reported or suspected. Overall, four decades of research suggests school-based CSA-prevention programs significantly improve CSA-related knowledge (Davis & Gidycz, 2000; Topping & Barron, 2009; Walsh et al., 2018; Zwi et al., 2007).

To realize the public health impact of school-based interventions, effective school-based CSA-prevention programs must be disseminated and implemented with fidelity on a wide scale. The current study describes the student-level effects of a large-scale implementation of *Safe Touches* in a Mid-Atlantic state. The intent was to demonstrate that it is possible to maximize the reach of a school-based CSA prevention program, and that the *Safe Touches* program can increase second grade students’ CSA-related knowledge and the increase can be maintained over time. The outcomes of interest were the reach (i.e., the number of students who receive the *Safe Touches* workshop) and effectiveness (i.e., the impact of *Safe Touches* on CSA-related knowledge over time; Glasgow et al., 2019). Results may be used to support and inform the future dissemination and implementation of this and other school-based CSA prevention programs on a wide scale.

## Method

The study used a longitudinal, within-group cohort design to demonstrate the reach and effectiveness of *Safe Touches* when implemented on a wide scale across four implementation sites (referred to hereafter as sites), representing five counties in a Mid-Atlantic state. All procedures were approved by the university Institutional Review Board.

### Intervention: *Safe Touches*

*Safe Touches* is an evidence-based CSA primary prevention curriculum delivered in kindergarten through third grade classrooms by two trained facilitators from an organization outside the school (e.g., victim service agency). In a single 50-minute interactive workshop, students learn to identify private parts of the body, the difference between safe and not-safe touches, and the distinction between secrets and surprises (Pulido et al., 2015). Students practice these body safety concepts through role-play scenarios with racially ambiguous puppets using four key steps: trust their feelings, say “no,” try to walk away, and tell an adult (Holloway & Pulido, 2018). Each student is given an activity booklet designed to reinforce concepts and facilitate discussion with caregivers at home.

### Reach

Reach reflects the total number, proportion, and representativeness of individuals who receive an intervention (Glasgow et al., 2019). Though students are the ultimate recipients of the intervention, in this context, reach must also account for classrooms and school districts. The goal was to reach all (100%) second-grade students in public elementary schools in four sites, during the 2018 to 2019 and 2019 to 2020 academic years. Based on publicly available census-level data, it was estimated that *Safe Touches* could reach approximately 17,000 second grade students if all classrooms in targeted school districts across the four sites participated.

#### Procedures

At study inception, sites secured letters of support from school district superintendents indicating a commitment to implementing *Safe Touches* in their district. This included agreeing to coordinate scheduling of workshops and research assessments, to help collect parent permission forms, and to facilitate follow-up assessments (i.e., tracking participating students into the next grade). Schools received $100 for every classroom that received the workshop. A *Safe Touches* trained facilitator at each site was then responsible for liaising with each school to schedule workshops and research assessments. Often, this facilitator (or organization) had an existing relationship with the school and had previously provided educational preventive programming. In other cases, the implementation of *Safe Touches* was the first CSA prevention program to be offered in the school or district.

The delivery of the workshop was distinct from research activities, as *Safe Touches* is a program that could be implemented outside of research. For this reason, procedures notifying parents of the workshop varied by school district ranging from implied permission to written opt-out. The university-based research team provided facilitators with a flyer to be sent home to all guardians with an overview of the workshop and notifying them that their child’s classroom would soon receive the workshop. The flyers were distinct from parent permission forms specific to the research. Students without permission to participate were given an alternative activity supervised by school staff. The number of second grade students who did not have parent permission to participate in the workshop is unknown.

#### Measures

*Safe Touches* facilitators at each site provided school-level information for the number of districts and second grade classrooms in which the workshop was delivered. Following each workshop, facilitators provided an estimated count of students in each classroom where they delivered the workshop. Because this estimate does not necessarily reflect the actual number of students per classroom, it is used as an *estimate* of the reach outcome for this study.

#### Analytic plan

In the current analysis, reach was operationalized as the number of children, classrooms, and school districts who received the *Safe Touches* workshop as reported by sites. Data are presented descriptively overall and by site.

### Effectiveness

Effectiveness reflects the impact of the intervention on the primary outcome of interest (Glasgow et al., 2019), defined here as children’s acquisition of CSA-related knowledge and the maintenance of that knowledge over time. Participants were second grade students who participated in the *Safe Touches* workshop, had parent permission to participate in research, and assented to participate in research activities.

#### Experimental procedures

Parent permission to participate in research was obtained by the university-based research team and was distinct from permission to participate in the *Safe Touches* workshop. In the weeks preceding the scheduled workshop, the research team distributed a flyer describing the research activities and a permission form to opt-in their child for participation in the research. The permission form was required to be signed and returned to the school for the student to be eligible to participate in the research. Students completed assessments at four timepoints: pre-workshop (T1; ~1 week prior to workshop), immediate post-workshop (T2), 6-months (T3) and 12-months post-workshop (T4). The COVID-19 pandemic and school closures in March 2020 precluded follow-up assessments among students that received the workshop in the Fall 2019. All workshops were delivered in-person, in the classroom.

At the first assessment (T1) a research team member obtained children’s assent to participate. As the assessment was administered, students who did not have parent permission to participate in the research or who did not assent were moved to a separate room where they worked quietly on their own work or were given an alternative activity at the teacher’s discretion. Students could be enrolled in the research any time up until the post-workshop survey. For example, a student may not have returned the form in time for the pre-workshop survey (T1), but could return the permission form the day of the workshop, agree to participate, and still be eligible to complete the post-workshop (T2) and follow-up surveys (T3 and T4). For completing the surveys, students received a university-branded pencil.

#### Measures

At the pre-workshop (T1) survey, children provided basic demographic information including age (open response) and gender (dichotomized as male or female). Supplemental school-level characteristics were extracted from publicly available data from the National Center for Education Statistics and the state Department of Education Office of Data Quality including percentage of students eligible for or receiving free-or-reduced meals and the racial composition (e.g., children identifying as American Indian, Asian, Black, Latinx, Native Hawaiian, or multiracial). These school-level characteristics were used as proxy indicators for student body composition: the percentage of students eligible for or receiving free-or-reduced meals was used as a proxy for income and the percentage of children identifying with a racial or ethnic minority status was a proxy for minority status. These school-level variables were grand mean centered and included as covariates in the analytic models.

The primary outcome of interest was children’s CSA-related knowledge. Survey questions were adapted from the Children’s Knowledge of Abuse Questionnaire (Tutty, 1995) which indicates children’s knowledge related to appropriate and inappropriate touches. Given constraints on classroom time, the assessment was comprised of six items reflecting those that indicated the greatest pre-post gains in prior research (Holloway & Pulido, 2018). Items relate to personal agency (e.g., “You have to let grown-ups touch you whether you like it or not,” “You always have to keep secrets,” “You can trust your feelings about whether a touch is good or bad”), what to do if a not safe touch happens (e.g., “It’s OK to say “No” and move away if someone touches you in a way you don’t like”), not safe touches from a familiar person (e.g., “Someone you know, even a relative, might want to touch your private parts in a way that feels confusing”), and appropriate touches (e.g., “A pat on the back from a teacher you like after you have done a good job at school is a safe touch”). The 6-item survey was answered on a three-point scale (2 = *true*; 1 = *in-between*; 0 = *false*) with higher scores indicating a greater knowledge. For analytic purposes, a mean score was used to create a composite knowledge score for each of the four assessment timepoints (T1–T4).

A secondary outcome of interest related to the effectiveness of *Safe Touches* was the number of disclosures made to facilitators by students during or immediately post-workshop. Adhering to University policies, facilitators notified the University when a disclosure was made that merited a report to the statewide CPS system. No identifiable information was provided to the University, only the date of the report made to CPSs. The University Department of Ethics and Compliance provided the total number of reports made by all facilitators throughout the implementation window.

#### Analytic plan

The analytic sample was limited to students with parental permission who assented to participate in research activities and who had opportunity to participate in all four assessments (*n* = 3,673). Multilevel modeling (MLM; Singer & Willett, 2003) was used to assess change over time in students’ level of CSA-related knowledge following the *Safe Touches* workshop. Level 1 reflected variability in children’s CSA-related knowledge over time, Level 2 incorporated child gender and accounted for variability between students, and Level 3 incorporated school-level characteristics (i.e., proxy variables for school socioeconomic level and racial/ethnic composition of student body) and accounted for variability between schools. All analyses were conducted using *xtmixed* in STATA Version 17 (StataCorp, 2019). A series of MLMs were tested. Model 1 (unconditional model with no covariates) estimated the intra-class correlation coefficients (ICCs) at the child and school levels. Model 2 (unconditional growth model) assessed the effects of including time as a predictor. To assess post-test and follow-up effects of the intervention on CSA-related knowledge, time was modeled as a categorical variable with T1 (pre-workshop) treated as the reference category. Model 3 included a child-level covariate (gender), and Model 4 included school-level covariates (income and minority status), which were grand mean centered to aid in interpretation of cross-level interactions with time. Follow-up contrasts were conducted to assess incremental changes in CSA-related knowledge over each subsequent time period using backward difference contrast coding.

## Results

### Reach

During the implementation period (2018–2019 and 2019–2020 academic years), *Safe Touches* was implemented in 718 classrooms across 58 school districts across the 4 sites (Table 1), reflecting 92% of all school districts. At Site D, there were three school districts that opted to not receive the *Safe Touches* workshop despite a signed letter of support from the superintendent. Approximately 14,235 second graders received the *Safe Touches* workshop over the 2018 to 2019 and 2019 to 2020 academic years. In the 2018 to 2019 academic year, the majority of students (75%; *n* = 5,989) received *Safe Touches* in the fall and one quarter of students (*n* = 3,395) received the program in the spring. The implementation of *Safe Touches* during the 2019 to 2020 academic year was directly impacted by the COVID-19 pandemic. At Site A, two engaged school districts who were scheduled for the *Safe Touches* workshop in March/April, but were not able to receive the workshop due to COVID-19 related school closures. Challenges notwithstanding, the *Safe Touches* workshop reached approximately 84% of the estimated second grade population across the four sites. Additionally, throughout the implementation period, no adverse events were reported.

**Table 1.** Reach of *Safe Touches* at the District, Classroom, and Student Level Across the Four Sites, Representing Five Counties in a Mid-Atlantic State.

	All Sites	Site A	Site B	Site C	Site D
Districts	58	11	13	24	11
Classrooms	718	245	253	92	128
Students	14,235	4,225	5,615	1,586	2,809

### Effectiveness

#### Sample

Among the students who provided demographic data (*n* = 3,639), most were 7 (67%) or 8-years old (32%). There was a nearly even distribution of students identifying as male (51%) and female (49%). School-level data, proxy variables for income and minority status, indicated the percent of students who were eligible for or received free-or-reduced meals, ranged from 3% to 100% and the percent of students who identified as a racial or ethnic minority group member ranged from 1% to 64% across all schools (see Table 2 for Mean % by site).

**Table 2.** Mean School-Level Proxy Variables for Minority and Income Status Overall and by Site.

	All Sites	Site A	Site B	Site C	Site D
	*GM* (%)	*M* (%)			
Racial or Ethnic Minority Group	31.2	29	23	28	44
Eligible for or Receiving Free-or-Reduced Meals	41.1	37	33	87	32

*Note. GM* = Grand Mean. *M* = Mean.

#### CSA-related knowledge

Model 1 provided estimates of the ICC at the child- and school-levels, indicating 6% of variability in CSA-related knowledge was attributable to between-school effects and 29% to between-child effects, with the remaining 65% of variability attributable to within-child effects. Time (Model 2) and gender (Model 3) effects did not differ substantively from those in the full model (Model 4); for simplicity, Table 3 presents only full model results. The final model revealed significant increases in CSA-related knowledge following participating in the *Safe Touches* workshop (γ_T2_ = 1.697, *p* < .001). Follow-ups 6- and 12-months post-workshop demonstrated continued evidence of higher levels of knowledge relative to baseline ratings (γ_T3_ = 1.354, *p* < .001; γ_T4_ = 1.477, *p* < .001). Follow-up contrasts revealed a small but statistically significant decline in CSA-related knowledge from T2 to T3, followed by a small but statistically significant increase in CSA-related knowledge from T3 to T4 (Table 4).

**Table 3.** Changes in CSA-Related Knowledge Attributed to the *Safe Touches* Workshop Among Second Graders (Final Model Results).

Parameter	Estimate (*SE*)	[95% CI]
Fixed Effects		
Intercept	7.526** (0.069)	[7.389, 7.663]
Time		
T2	1.696** (0.057)	[1.585, 1.808]
T3	1.354** (0.064)	[1.228, 1.479]
T4	1.477** (0.078)	[1.322, 1.632]
Gender (Female)	0.277** (0.073)	[0.135, 0.419]
Time × Gender		
T2	0.088 (0.078)	[−0.648, 0.242]
T3	0.137 (0.082)	[−0.024, 0.298]
T4	0.148 (0.094)	[−0.036, 0.332]
School-Level Effects		
% Low income	−0.092** (0.028)	[−0.146, −0.038]
Time × Income		
T2	−0.049** (0.191)	[−0.087, −0.012]
T3	−0.031 (0.022)	[−0.076, 0.013]
T4	0.059* (0.029)	[−0.002, 0.115]
% Racial or ethnic minority status	−0.010 (0.032)	[−0.074, 0.054]
Time × Racial or Ethnic Minority		
T2	0.112** (0.024)	[0.064, 0.160]
T3	0.018 (0.028)	[−0.037, 0.076]
T4	−0.038 (0.036)	[−0.109, 0.033]
Random Effects		
Level 1 (Time)	2.383 (0.045)	[2.296, 2.474]
Level 2 (Student)		
Variance—Time	0.119 (0.019)	[0.086, 0.166]
Variance—Between students w/in same school	1.911 (0.097)	[1.730, 2.110]
Level 3 (School)		
Variance—Time	0.017 (0.006)	[0.008, 0.033]
Variance—Between schools	0.234 (0.053)	[0.150, 0.365]

*Note*. Income is represented by the proportion of students at a school eligible for or receiving free-or-reduced meals. T1 = pre-workshop; T2 = immediate post-workshop; T3 = 6-month post-workshop; T4 = 12-months post-workshop.

* *p* < .05. **p < .001.

**Table 4.** Time Contrasts.

Total Score × Time	Contrast (*SE*)
T2 vs. T1	1.741* (0.0412)
T3 vs. T2	−0.319* (0.0423)
T4 vs. T3	0.129* (0.049)

* *p* < .001.

**Figure 1.**
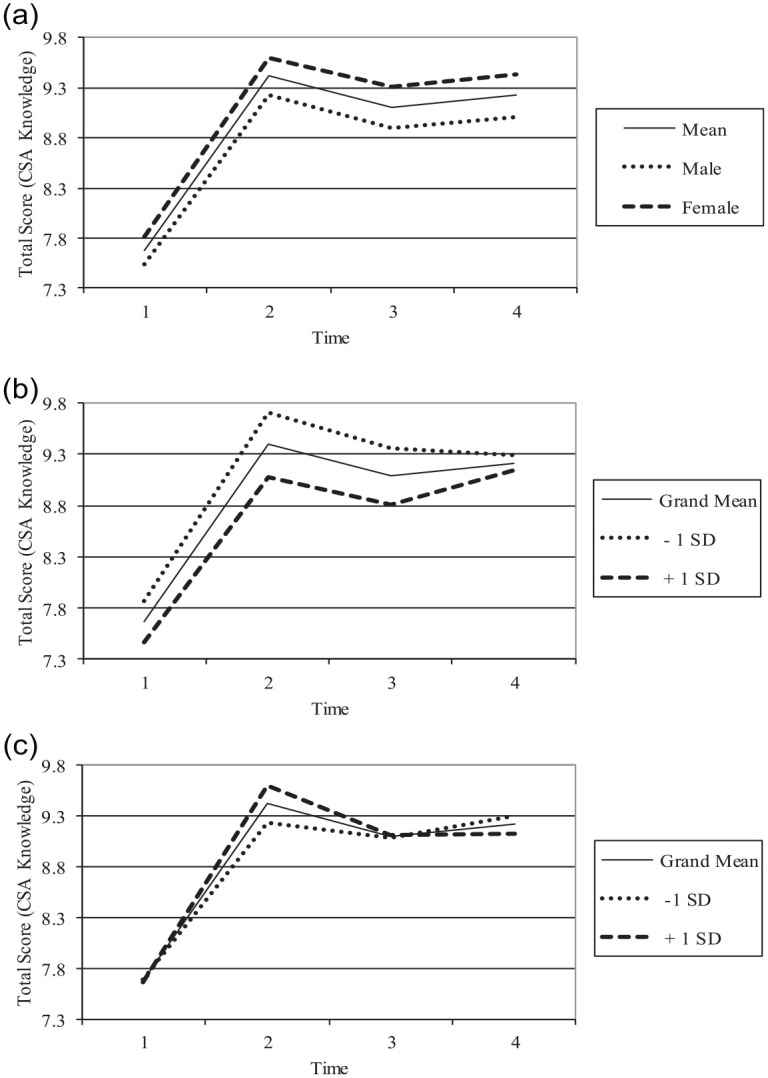
(a) Time × Gender interaction, (b) Time × Income status interaction, and (c) Time × Minority status interaction. *Note*. Figure 1 reflects margins estimated from the final model, conditioned on model covariates. Figure 1a depicts the average marginal effect of gender on CSA-related knowledge, controlling for school-level income and minority status. Figure 1b depicts the average marginal means for CSA-related knowledge based on income, as measured by the percentage of students eligible for free and reduced meals (±1 SD) at the school level, controlling for gender and minority status. Figure 1c depicts the average marginal means for CSA-related knowledge based on minority status, as measured by the percentage of children identified as minority at the school level (±1 SD), controlling for gender and income. CSA = Child sexual abuse.

Child and school-level covariate effects are summarized in Table 2 and depicted in Figure 1. Female CSA-related knowledge was significantly higher than male ratings at T1 (γ_Female_ = .277, *p* < .001), with no evidence of a gender-by-time interaction (Figure 1a). Participants in schools with a greater percentage of low-income students (i.e., those receiving free-or-reduced meals) had lower ratings of CSA-related knowledge at baseline (γ_%Income_ = −.092, *p* = .001); these effects varied as a function of time, though these effects largely disappeared by T4 (Figure 1b). The school-level percentage of minority students did not affect baseline CSA-related knowledge scores (γ%_Minority_ = −.010, *p* = .760). There was a small but statistically significant interaction over time indicating a steeper rate of increase in knowledge among students in schools with a greater percentage of minority students, but this interaction was not maintained over time (Figure 1c).

#### Disclosures

A secondary signal of the effectiveness was disclosures made during the workshop or research procedures; however, this must be interpreted with caution as there was no control group in the design. During the full implementation period, 29 disclosures were made to facilitators during or following the workshop that warranted a report to the CPS system. Any reports made to school officials, mandated reporters, or other safe adults would not be represented in this count. Anecdotally, from conversations with the facilitators, we know that other reports were made following the workshop that specifically referenced *Safe Touches*.

## Discussion

A major objective of this study was to demonstrate the feasibility of achieving maximum reach of a CSA primary prevention program for second graders. In this effort, this implementation trial was able to reach students in 92% of the school districts approached across four sites reflecting urban/rural, low/high income, and racially diverse settings. This effort reached over 14,000 second grade students in a total of 718 classrooms within two academic years. To our knowledge, this reach represents the largest population to receive a universal school-based CSA prevention program in a single implementation. Some sites were able to deliver the *Safe Touches* workshop in 100% of school districts while other sites were less successful (Table 1). Reasons for non-participation included interruptions by the COVID-19 pandemic (Site A) and a commitment to a non-evidence-based locally developed program (Site D). School closures to mitigate a global pandemic could not have been anticipated, but important lessons may be learned from those few districts who chose to implement a program not meeting evidence-based criteria. For example, *Safe Touches* facilitators anecdotally shared that the letters of support from Superintendents were beneficial in arranging programming with counselors. Thus, future endeavors might invest more time in helping school administrators understand the benefit of selecting evidence-based programming. Other factors that potentially contributed to the reach of the *Safe Touches* workshop include the use of external facilitators (i.e., not asking school staff to do additional programming) and the classroom incentive. Nevertheless, the reach achieved by this implementation trial signals that diverse school districts will indeed sign on to CSA prevention for elementary school students, that schools will devote classroom time to such implementation, and a high number of students can be reached across different contexts in a short period of time.

A second objective was to demonstrate the effectiveness of *Safe Touches* in a longitudinal cohort design. Results indicate a significant increase in CSA-related knowledge following *Safe Touches* and that this increase was maintained at 12-months post workshop. Although there was a significant decrease in CSA-related knowledge gains between post-workshop and the 6-month assessments, CSA-related knowledge at 6-months was significantly higher than baseline and the pre- to post-workshop gains were recouped by the 12-month follow-up. The reason for the increased effectiveness at the 12-month assessment is not clear, but could be attributed to the fact that students completed this follow-up assessment in third grade and may have received additional prevention programing (e.g., bullying prevention, school shooting workshops) and/or been exposed to additional sources of information pertaining to CSA prevention and psychosexual development.

Multilevel models indicated some pre-workshop level differences in CSA-related knowledge. Specifically, at pre-workshop, females scored higher than males whereas students in schools with a higher percentage of low-income had significantly lower levels of CSA-related knowledge. However, there were no statistical differences on covariates at the 12-month assessment. Despite females demonstrating a significantly higher level of knowledge than males pre-workshop, there was no gender by time interaction indicating that, although females knew more initially, males acquired knowledge at the same rate as did females. The reason for higher knowledge among females is not entirely clear, but social learning theory of gender development has long suggested that females receive earlier and different sex-related education compared to their male counterparts (Perry & Bussey, 1979). School-level covariates (income and minority status) were extracted from publicly available datasets and do not necessarily reflect the students who participated in the research; however, a lack of significant differences at 12-months suggests that students across different contexts are able to learn and retain CSA-related knowledge following the *Safe Touches* workshop.

Given that the majority of extant research has not reported on the potential for long-term maintenance of CSA-related knowledge in pediatric samples, the 12-month follow-up results are a notable strength of this study. However, there are a few limitations to the results presented. The absence of a comparison group limits causal conclusions about the effectiveness of the program, but this was not the goal of the research team as the effectiveness of *Safe Touches* was previously established (Holloway & Pulido, 2018; Pulido et al., 2015). The lack of a control group also hinders the interpretability of the disclosure data. Specifically, without a control group we cannot determine conclusively that these disclosures would not have happened elsewhere in the absence of the program. Future research could examine school-level disclosures in other years. While it is clear that the *Safe Touches* workshop resulted in disclosures, this study did not assess behavior change which would be an important step for determining whether there is a relationship between children’s knowledge and future victimization. Further, it is not clear that students could or would retain this knowledge over a longer term or as they develop into the middle school years and encounter developmentally salient experiences regarding sexual development (e.g., at the onset of puberty). Future research should consider attempts to follow participants over a longer period to ascertain whether they can retain knowledge throughout multiple developmental phases and contexts as well as whether the *Safe Touches* workshop might result in the use of protective behaviors over time (e.g., disclosure of attempted or completed victimization to safe adults) and, ultimately, whether use of protective behaviors by children impacts their victimization (e.g., by deterring victimization attempts).

Findings presented offer empirical evidence in rebuttal of the main criticisms of universal, school-based CSA prevention such as the age-appropriateness of content, and potential harmful side effects for children (see discussions in Finkelhor, 2009; Kenny, 2010; Wurtele, 2009; Zeuthen & Hagelskjær, 2013). Results presented here suggest the contrary; that second-grade students, mostly 7-years old, can significantly increase their CSA-related knowledge and retain that knowledge 1-year post workshop. Moreover, CSA prevention can be delivered to children without adverse effects. Being that this was an implementation trial with Human Subjects protections and oversight, the reporting of adverse events was required. That none were reported from providers, educators, parents, or children suggests the prevailing acceptability of the CSA prevention content.

Another criticism of school-based CSA prevention programs is the lack of effect on disclosures (or rates of CSA). In our study, 29 unique disclosures were made during or following the workshop perhaps hastening the end of abuse and initiating CPS intervention. Though not a direct assessment of the impact that *Safe Touches* may have had on overall rates of CSA, the reduction in the duration or severity of abuse following disclosure intuitively reduces the lifetime impact and therefore the economic burden of CSA. As described, the large-scale implementation of a universal school-based CSA prevention program is indeed possible and may offer meaningful public health impact. However, children should not bear the responsibility for preventing their own abuse. Thus, to maximize this impact, school-based programs could be implemented in conjunction with other modalities and targets of CSA prevention namely efforts engaging parents (Guastaferro et al., 2022; Mendelson & Letourneau, 2015; Rudolph et al., 2018), those designed for older children (Letourneau et al., 2017; Ruzicka et al., 2021), those focused on perpetration prevention (Assini-Meytin et al., 2020; Stephens et al., 2022), and those that are implemented for adults in the larger community (Assini-Meytin et al., 2021). Coordinated, multi-pronged prevention efforts hold the greatest promise for realizing public health impact and producing a change in rates of CSA.
